# Tribological Characterisation and Modelling for the Fused Deposition Modelling of Polymeric Structures under Lubrication Conditions

**DOI:** 10.3390/polym15204112

**Published:** 2023-10-17

**Authors:** Feiyang He, Chenyan Xu, Muhammad Khan

**Affiliations:** 1 Centre for Life-Cycle Engineering and Management, Cranfield University, Cranfield MK43 0AL, UK; feiyang.he@cranfield.ac.uk; 2 School of Aerospace, Transport and Manufacturing, Cranfield University, Cranfield MK43 0AL, UK; chenyan.xu.610@cranfield.ac.uk

**Keywords:** 3D printing, ABS, PC, PLA, coefficient of friction, wear rate, lubrication, infill density, layer thickness

## Abstract

In recent years, additive manufacturing technology, particularly in plastic component fabrication, has gained prominence. However, fundamental modelling of the influence of materials like ABS, PC, and PLA on tribological properties in fused deposition modeling (FDM) remains scarce, particularly in non-lubricated, oil-lubricated, and grease-lubricated modes. This experimental study systematically investigates the effects of material type, lubrication method, layer thickness, and infill density on FDM component tribology. A tribology analysis is conducted using a TRB3 tribometer. The results indicate a coefficient of friction (CoF) range between 0.04 and 0.2, generally increasing and decreasing with layer thickness and filler density. The lubrication impact hinges on the material surface texture. The study models the intricate relationships between these variables via full-factor analysis, showing a strong alignment between the modelled and measured friction coefficients (an average error of 3.83%). Validation tests on different materials affirm the model’s reliability and applicability.

## 1. Introduction

Fused deposition modelling (FDM) is one of the vital additive manufacturing technologies for polymeric materials. It uses raw material in the form of solid filament. The filament is heated to a molten state in an extrusion chamber. Afterwards, the molten filament is extruded from a nozzle onto a platform where it is cooled and solidified [1]. It has the advantages of low manufacturing cost, easy operation, and efficient use of materials. However, it also has disadvantages, such as poor mechanical properties and low surface quality.

To mitigate these disadvantages, extensive studies have investigated and tried to improve the mechanical performance of FDM parts. Most of them have focused on two main areas: the printing materials and forming parameters [2,3,4], since various materials and forming parameters, such as nozzle size, layer thickness, and infill density, have a complex impact on their mechanical properties [5]. Numerous studies have investigated their static mechanical properties, including elastic modulus and tensile strength [3,6]. Meanwhile, some studies have investigated their dynamic mechanical properties, including fatigue strength and vibration [7,8]. In addition, some research also explored the tribological properties of the high potential of FDM structures in transmission systems such as gears [9].

Some of them have evaluated the friction behaviours of FDM polymers against injection-moulded samples [10,11]. In Amiruddin et al.’s study, FDM and moulded ABS samples were tested under oil lubrication conditions. The experimental results yielded that the FDM sample has a more significant CoF (0.040~0.055) compared to the moulded one (0.009~0.025) [10].

On the other hand, some studies have focused on the material’s influence on the FDM polymer’s tribological properties. Hanon et al. measured the CoF for FDM PLA and ABS. The results had no significant difference for the two materials [12]. They also tested the CoF for FDM nylon and a kind of novel polymer matrix, making new discoveries about the tribological properties of lubrication [13,14]. Another body of research made a more comprehensive investigation with different conclusions. It tested FDM PCL, ABS, PLA, and co-polyester. The results showed that the PLA and co-polyester samples had the lowest friction coefficients, while the ABS and PCL samples had the most significant test coefficients of friction [15]. Cardoso et al. added carbon black (CB) and alumina (ALM) nanofillers to the FDM PLA filament to improve the tribological properties of the FDM polymers. The nanocomposites containing 25 wt% ALM and 75 wt% CB presented better wear properties with evidence of increased viscosity [16]. Similarly, Pan et al. compared the wear rate of pure and carbon nanotube (CNT)-reinforced FDM PPS samples. Compared to pure PPS, the wear rate of the CNT-reinforced piece was reduced from 1.14 × 10^−3^ mm^3^ N^−1^ m^−1^ to 1.838 × 10^−5^ mm^3^ N^−1^ m^−1^ [17].

The above studies all emphasise the material’s influence. However, the complex forming parameters also significantly affect the tribological behaviours. Akıncıoğlu et al. printed gyroid-patterned ABS samples with different infill densities (25%, 50%, and 75%) and investigated their wear properties. The tribological test results concluded that the samples with 75% infill density had the highest CoF [18]. Similarly, Perepelkina et al. conducted the same investigation on FDM PLA samples. They came to the same conclusion that higher infill density increased the CoF [19].

In addition to the infill density, other studies have evaluated several printing parameters such as printing pattern, raster angle, layer thickness, nozzle temperature, plate temperature, and printing speed [4,20,21,22,23,24,25,26,27]. Moreover, two of them provided empirical models based on experimental data. One body of research analysed the dependent variables, CoF, and wear rate, as a function of the nozzle temperature, layer height, and printing pattern for FDM ABS. The developed empirical model suggested that layer height affects the CoF and wear rate most significantly [21]. Likewise, Hervan et al. used a linear regression model to correlate the friction coefficient, layer thickness, and layer orientation for FDM PLA [22].

The above review showed some previous work on FDM polymer tribology. Most of the investigations were still limited to material characterisation with different materials and printing parameters. Only two studies proposed bespoke empirical models for specific materials. They cannot be applied to all other FDM polymers, significantly limiting their significance. A generalised model that introduces the influence of different materials and printing parameters on tribological properties must be improved in current academia. Furthermore, only one body of research considered lubrication during the tests. However, a lubricated environment is familiar and essential in actual working conditions. Therefore, it is vital to investigate the tribological properties of specimens under different lubricated conditions.

To fill these gaps, the presented research investigated and modelled the relationships between the friction properties, materials, and printing parameters under different lubrication conditions. Parametric tribological experiments were conducted to determine the friction-related performance indicators (the friction coefficient and wear rate). The research-tested ABS, PC, and PLA samples with different printing parameters (layer thickness and Infill density) under three lubrication conditions (dry, grease, and oil). The experimental results were analysed and used to develop an empirical model. Then, the model was validated by the tribology test using a PC-ABS sample with arbitrary parameter selections. The experimental results and proposed model can help to predict and improve the friction performance of 3D-printed products in real applications.

## 2. Materials and Methods

### 2.1. Materials Selection

The research selected the ABS, PC, and PLA from Ultimaker^®^, Geldermalsen, The Netherlands, and the PC-ABS blend from RS-PRO, the UK, as the raw materials. ABS has good all-around properties and is inexpensive. It is the most common material used in the FDM industry [28]. PC is a polymer with carbonate groups in its molecular chain. It has good mechanical properties, and it has better wear resistance compared to ABS material [29]. PLA is a new type of biodegradable material made from starch raw materials raised from renewable plant resources such as maize. It is thermally stable and can be adapted to lower printing temperatures [30]. PC-ABS is a new blend, an engineering-grade material often used in industrial applications, with excellent toughness and heat resistance and a good surface finish for 3D-printed parts [29], so the research selected it as the validation material. The filament diameter used in this study was 2.85 mm, and the specific specifications are shown in Table 1.

### 2.2. Specimen Preparation

**Figure 1:**
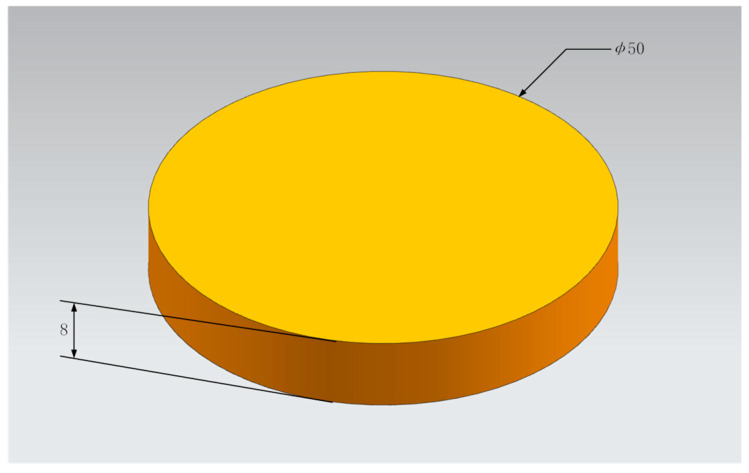
Specimen geometry.

The specimen’s geometry is shown in Figure 1. It is a disc with a diameter of 50 mm and a thickness of 8 mm, which meets the requirements of a tribological test bench. The model designed in UGNX^®^ was saved in STL format and imported into UltiMaker Cura^®^ 5.0 software for slicing and setting up the printing parameters. The software generated G-code files that the Ultimaker 2+ printer read for printing. Except for the printing parameters to be evaluated, all the parameters were set as the default recommended values to ensure sample quality. Some standard parameters are shown here: Nozzle diameter: 0.8 mm; Wall line count: 2, Infill pattern: Grid, Print speed: 40 mm/s, Top/Bottom sickness: 1.2 mm. A grid infill pattern was selected because it provides a good balance between strength and material conservation.

### 2.3. Experimental Scheme

Three parameters (layer thickness, infill density, and lubrication type) were chosen to investigate their influence on the friction characteristics. Two of them are process parameters. The layer thickness refers to the height of each stacked layer during the 3D-printing process. Its variation directly impacts the surface properties of the printed specimen, consequently affecting the surface friction characteristics [25]. The infill density, on the other hand, determines the amount of material filling within the specimen space, influencing the strength, weight, and printing time of the 3D-printed object. The lubrication is the test parameter. The lubricants change the friction between the two frictional objects, reduce wear, and extend life.

Each parameter was set to three levels, as shown in Table 2. In Ultimaker Cura, the default recommended parameters of the software are 0.2mm layer thickness and 50% infill density. Therefore, layer thicknesses of 0.2, 0.25, and 0.3 mm were used in this study, covering the range of standard settings for printing rough to precision products. 25, 50, and 75% were selected as different infill densities. The lubrication modes were classified as no lubrication, grease lubrication, and oil lubrication to represent common lubrication conditions.

The complete factorial experiment method was applied to comprehensively evaluate each parameter’s influence on the tribological properties. That meant there were 81 combinations. The test was repeated once for each variety to ensure the repeatability of the experimental results.

### 2.4. Experimental Setup and Procedure

**Figure 2:**
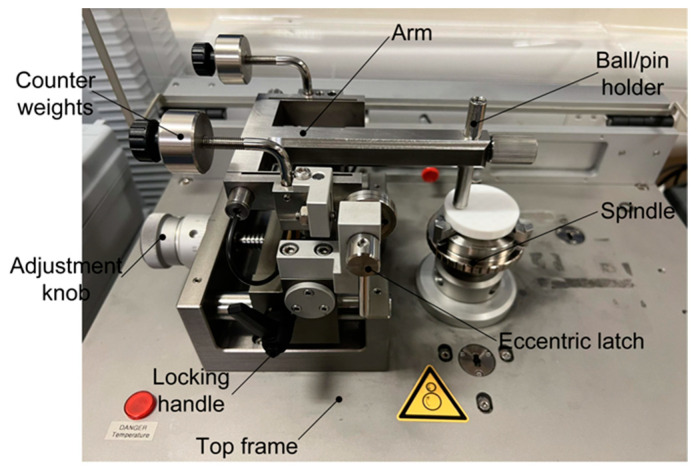
Experimental setup.

The experiment used the TRB^3^ from Anton Paar, a pin-on-disc tribometer (Figure 2), for the tribological tests. It can effectively depict the tribological behaviours using a simple disc movement and measure the CoF and wear rate of the material.

Before starting the experiment, the specimens were thoroughly dried. Afterwards, the model was fixed on a three-jaw chuck. An eccentric small ball of stainless steel (with a hardness of 223 HB and Young’s modulus of 194 GPa) was set to the arm and kept perpendicular to the disk with a 15 mm wear track radius. A constant 10N load was applied to the ball, and the disk was rotated at 300 revolutions per minute. So, the ball slides against the specimen during the operation. A total of 1 min of friction cycling was performed at a room temperature of 25 °C and under the lubricated conditions in the scheme.

To prevent the polymeric material from being left on the pellets after the friction test, the surface of the shots was cleaned with ethanol before and after each test. The program that comes with TRB^3^ automatically calculates the wear rate of the specimen during the experiment. It is determined by calculating the loss of volume. As the stiffness of the specimen material differs significantly from that of the stainless steel spheres, the wear test assumes that only the discs are worn. The volume loss of the disc at this point can be calculated using the following Equation (1).

(1) $$V_{disk}=2\pi R\left[r^{2}\sin^{-1}\left(\frac{d}{2r}\right)-\left(\frac{d}{4}\right){\left(4r^{2}-d^{2}\right)}^{\frac{1}{2}}\right],$$

where *R* is the wear track radius, *d* is the wear track width, and *r* is the ball radius.

Once the disc volume loss $V_{disk}$ has been obtained, the wear rate of the disc $W_{disk}$ can be calculated, which is usually expressed in $m^{2}/N$ using the equation in Equation (2).

(2) $$W_{disk}=\frac{V_{disk}}{F_{n}l}$$

where $F_{n}$ is the normal phase load, and l is the total friction distance. In addition to comparing the wear rates, this study also compared the variation in the CoF for different parameters. The CoF is the ratio of the frictional force F to the applied load $F_{n}$. During the experiment, the software equipped with TRB^3^ automatically displays the friction force and CoF for each specimen during the investigation. However, this value is constantly changing so that in the final calculation, the friction force of the model is expressed as its mean.

### 2.5. Experimental Model Development

To obtain an experimental model of the effect of the 3D-printing materials and parameters on tribological properties, the research established the expressions of the transfer function of the output quantities (friction coefficient and wear rate) against the input quantities (layer thickness, filling density, lubrication method, and material) using MATLAB^®^ (MathWorks, Natick, MA, USA). The data obtained from the tests were imported into MATLAB^®^ to fit into the regression model automatically. This study uses one of its properties to quantify the material and lubrication type parameters as the basis for modelling.

When a small ball rubs a disc specimen, its surface is compressed and gradually peeled off. Therefore, a higher shear modulus means that the surface will wear more slowly, i.e., the wear rate will be lower [31,32]. Therefore, this study uses the material’s shear modulus as the basis for modelling.

The primary function of a lubricant is to form a lubricating film between contact surfaces to reduce direct contact and friction. The lubricant’s viscosity directly affects the lubricating film’s formation and stability. Too low a viscosity may result in a weak lubricating film that is ineffective in reducing contact and friction. At the same time, too high a viscosity may increase adhesion and sticking forces, leading to increased friction [33]. Therefore, this study uses the viscosity of the lubricating fluid as the basis for modelling.

### 2.6. Model Calibration

Once all the experimental data were input into MATLAB, it was possible to generate an empirical model for the CoF and wear rate considering the layer thickness, infill density, lubrication conditions, and materials. To validate the robustness of the model, the study repeated the parametric experiments utilising PC-ABS materials. The obtained data were then compared against the model’s predicted values to assess the reliability of the experimental model for different printing parameters, materials, and lubrication settings.

## 3. Results and Discussion

### 3.1. Effect of Independent Variables on the CoF

**Figure 3:**
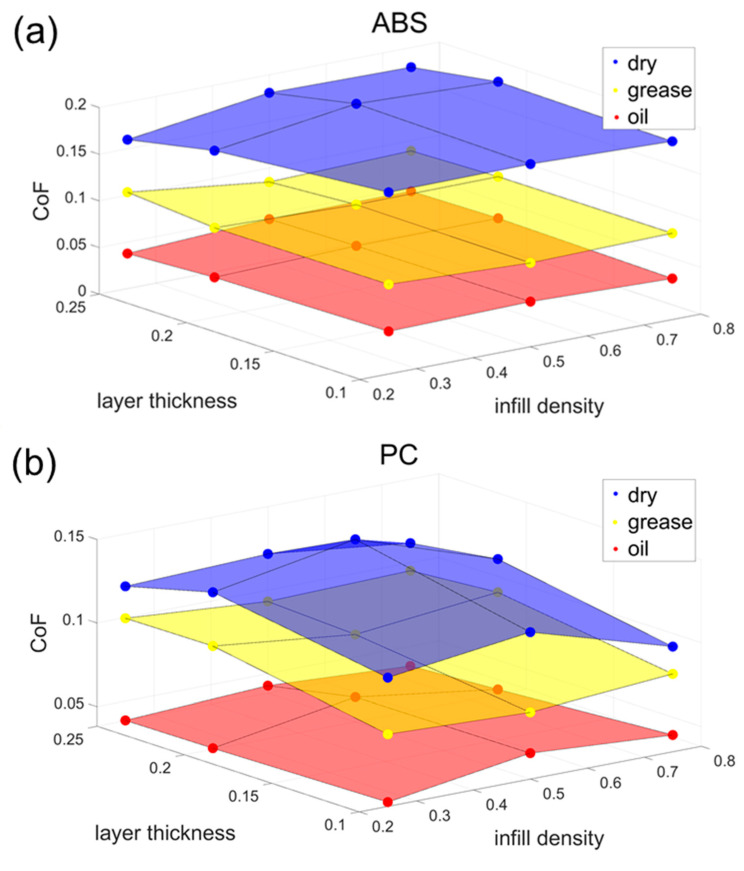

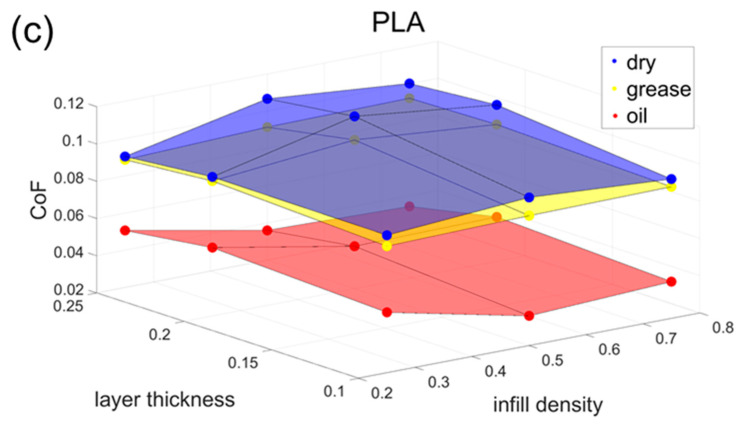
Experimental CoF results. (**a**) ABS. (**b**) PC. (**c**) PLA. Layer thickness unit: mm. Coloured dots represent the experimental data.

Figure 3 shows the test results of the CoF for the three materials, ABS, PC, and PLA, under different lubrication modes with various infill densities and layer thicknesses. The different colours indicate the lubrication conditions. The highest CoF values were obtained using a layer thickness of 0.2 mm and a fill density of 50%, regardless of whether the material tested was ABS, PC, or PLA.

The three distinct colourful layers suggest that the lubrication conditions have the most significant influence on the CoF. All the materials had similar and the lowest CoF values, around 0.05, under the oil lubrication conditions. After the grease lubrication, the CoF for the three materials increased to approximately 0.1. The highest CoF values occurred under dry conditions, and it is worth noting that compared to the other lubrication conditions, the three materials’ CoF values here appear different. ABS has the highest CoF value, 0.16, followed by 0.13 for PC. PLA has the lowest CoF value, 0.11. This is because PLA has the smoothest surface under the same printing parameters with almost no print texture. The differences between the three materials are most pronounced without lubrication, and once lubrication is present, the differences between the three materials are significantly reduced.

**Figure 4:**
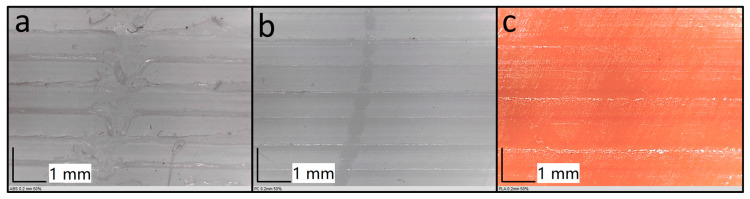
Specimen top surface and wear track for (**a**) ABS. (**b**) PC. (**c**) PLA with 0.2 mm layer thickness and 50% infill density.

Regarding the CoF differences under grease conditions, the main reason affecting the lubrication sensitivity is the different surfaces of 3D-printed polymers. The typical print surfaces and wear tracks of the three materials are shown in Figure 4. PLA has the smoothest texture for the same print parameters, while both PC and ABS have grooves on the surface caused by the print path [34,35,36]. Both PC and ABS have grooves on the surface caused by the print path, where PC is depressed downwards, and ABS is raised upwards. This leads to the fact that in grease-lubricated conditions, the surfaces of PC and ABS can hold some of the greases in these grooves to achieve lubrication, whereas the smooth surface of PLA cannot.

In most cases, it is observed that the CoF for 3D-printed specimens exhibits a pattern of initially increasing and subsequently decreasing as the infill density is adjusted. An exception to this trend is noted in the case of PLA specimens under grease-lubricated conditions. Furthermore, the variance in the CoF with respect to the infill density is most prominent when specimens are devoid of lubrication, whereas the CoF values exhibit relatively minimal fluctuations under the other two lubricated conditions.

When the infill density is set at a relatively low level, the surface of the specimen tends to manifest small undercuts owing to the presence of grid-filled voids. These undercuts give rise to a transient overhang of the tribometer pin as it traverses this region, leading to an abrupt reduction in the contact pressure experienced between the pin and the specimen’s surface. This phenomenon results in a momentary decline in the CoF, contributing to a diminished average value in the final calculation. Conversely, when the infill density is raised sufficiently, the specimen exhibits fewer internal voids and possesses an enhanced surface-bearing capacity [25]. Consequently, the surface quality of the model improves, resulting in a smoother surface with a reduced surface roughness and a lowered CoF.

The variation in layer thickness exerts a relatively modest influence on the alteration of the CoF, albeit maintaining the overarching trend of an increase followed by a subsequent decrease as the layer thickness is augmented.

When the uppermost layer’s thickness is set at a minimum threshold of 0.1 mm, the material extrusion becomes notably compact, thereby augmenting the load-bearing capacity of the topmost layer in the specimen. This phenomenon, in turn, results in a marginal reduction in the extrusion depth of the pin, consequently leading to a diminishment in the CoF [27]. Conversely, when the thickness of the uppermost layer surpasses a certain threshold, the creation of fine grooves within the layer provokes a momentary suspension of the tribological wear test needle as it traverses this region. This suspension event precipitates an abrupt reduction in the contact pressure between the pin and the specimen surface [22], eliciting a transient decline in the CoF. This decline ultimately contributes to a reduction in the final calculated average value.

### 3.2. Effect of Independent Variables on Wear Rate

**Figure 5:**
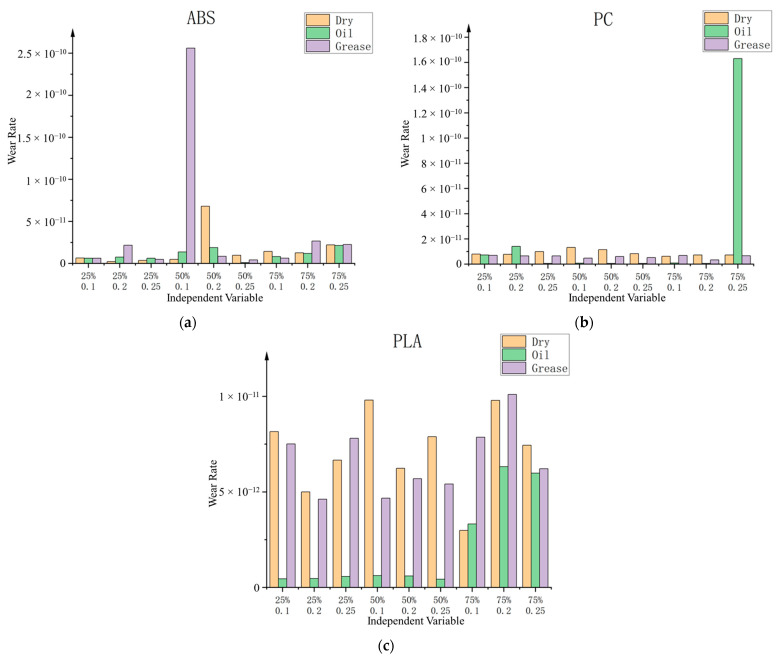
Experimental wear rate results. (**a**) ABS. (**b**) PC. (**c**) PLA. Wear rate unit: m^2^/N. Independent Variables: First row: Infill density. Second row: Layer thickness (mm).

The wear rate of the specimen surface can be calculated from Equations (1) and (2). The results are visually depicted in Figure 5. Broadly, it is observed that under most oil lubrication conditions, the wear rate exhibits a lower magnitude when compared to a non-lubricated state. Conversely, in contrast, in the case of grease lubrication, while the wear rate is reduced, the magnitude of this effect is not significant.

During this investigation, it was observed that the tribological experiments conducted, characterised by relatively modest loads and brief durations, did not yield readily discernible wear marks on the surface of the specimens. Instead, the wear rate calculation was predicated on the assessment of wear depth, as gauged by the TRB3 measuring apparatus. This approach introduced a significant degree of deviation into specific experimental outcomes.

**Figure 6:**
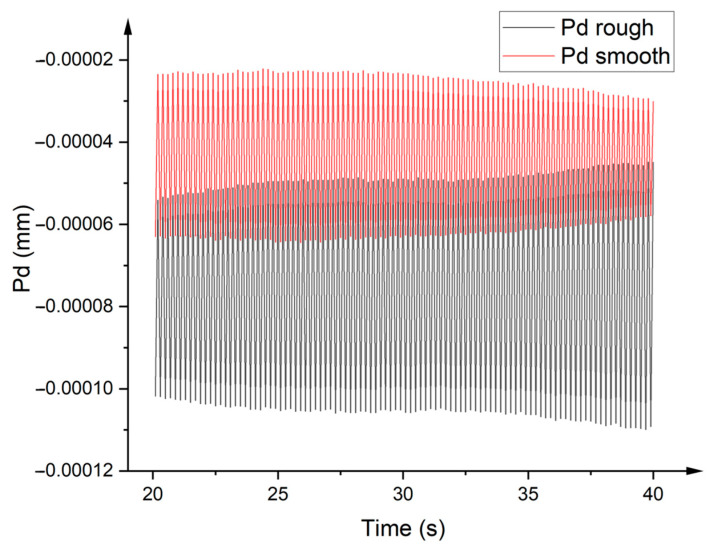
Depth variation of different surfaces during testing.

The principal source of this observed deviation in the study stems from disparities in the surface texture of the specimens under examination. As depicted in Figure 6, when the specimen surface exhibits a more pronounced printed texture, it manifests a heightened surface roughness and an amplified amplitude in the depth curve. This amplified roughness imparts a greater vibrational amplitude on the measuring device when ascertaining the surface height, leading to instances where the measurements deviate markedly in magnitude, oscillating between values that are either excessively elevated or considerably diminished.

In the context of other experimental findings, which exhibit a notable absence of significant deviations, it becomes apparent that the wear rate in oil lubrication instances is markedly reduced compared to the other two lubrication modalities. In certain cases, this reduction reaches orders of magnitude. Conversely, the wear rate observed during grease lubrication manifests minimal disparity from that of the non-lubricated specimen. This phenomenon can be attributed to the observed tendency of grease to be displaced from the wear region by the rotating pin during the disk’s motion, resulting in surface wear akin to that observed in the absence of lubrication.

In a broader analysis, alterations in layer thickness are found to exert a marginal influence on the specimen’s surface wear. Conversely, an elevation in filler density is causally associated with an increase in the wear rate. This study posits that this relationship arises due to the heightened filler content, contributing to a smoother and more supportive surface texture on the specimen. Consequently, this enhanced surface quality facilitates a more comprehensive and extensive interaction between the pin and the specimen, leading to increased friction and subsequent wear.

## 4. Empirical Model Development and Validation

**Figure 7:**
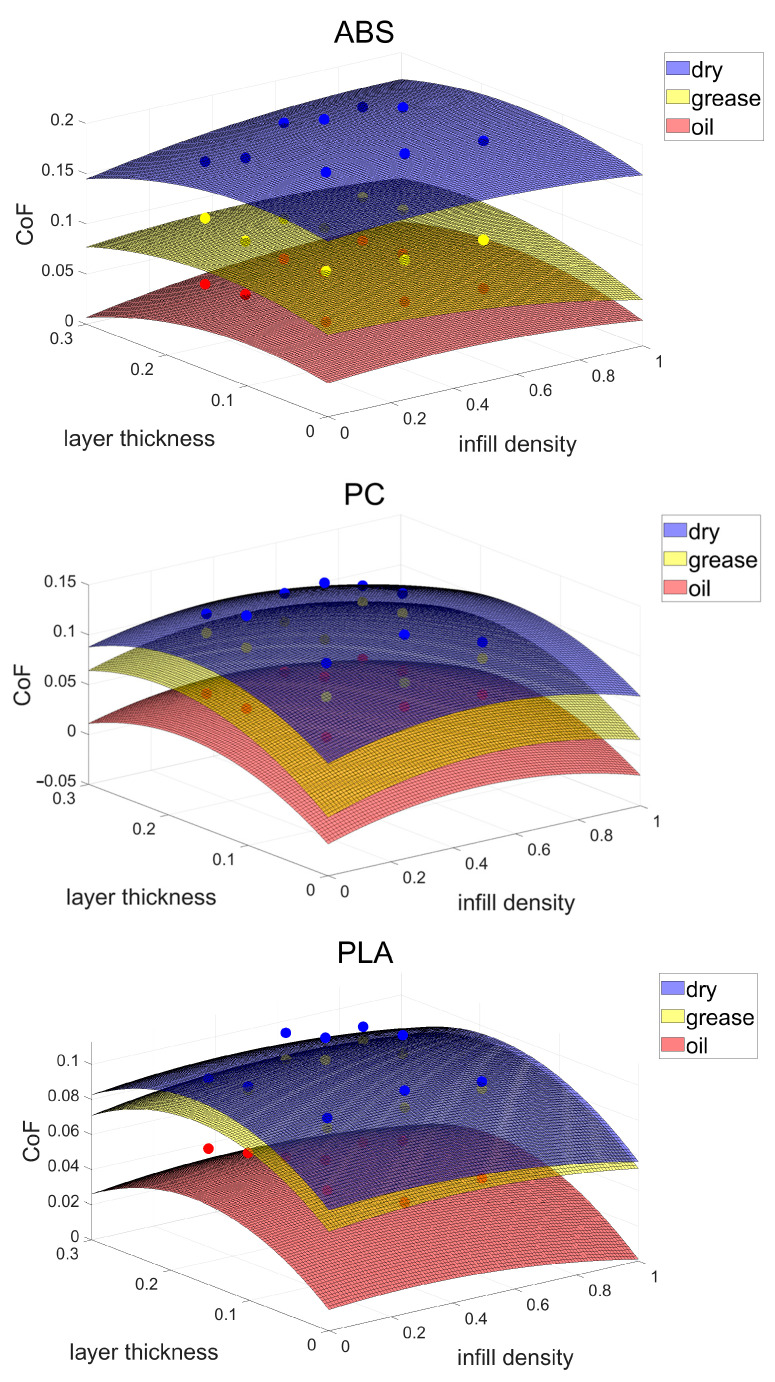
Empirical CoF model surfaces. Layer thickness unit: mm. The coloured dots represent the experimental data.

The data presented in Figure 3 reveal a non-linear relationship between the CoF results, layer thickness, and infill density for the three materials under investigation. Given that each independent variable influences the experimental outcomes, it is imperative to acknowledge that alterations in different independent variables collectively impact the CoF of the specimen’s surface. Consequently, when selecting a nonlinear polynomial function for modelling using MATLAB^®^ R2019b, it becomes essential to account for the simultaneous influence of diverse independent variables on the ultimate results. The formula chosen for this research is shown in Equation (3).

(3) $$CoF=p_{000}+p_{001}\times \mu +p_{010}\times I+p_{100}\times L+p_{110}\times \mu \times I+p_{011}\times I\times L+p_{101}\times \mu \times L+p_{002}\times \mu^{2}\;+p_{020}\times I^{2}+p_{200}\times L^{2}$$

where μ is the viscosity, I is the infill density, and L is the layer thickness. Figure 7 shows the variation in the CoF values for different materials under different lubrication conditions. The faces in the figure represent the surface of the fitted function for that condition, while the dots represent the CoF results obtained from actual tribological experiments.

Where the fitting equation for ABS is

$$CoF=0.17417-0.12322\times \mu +0.029071\times I+0.15498\times L-0.0031232\times \mu \times I+0.0084987\times I\times L\;+0.11072\times \mu \times L+0.011392\times \mu^{2}-0.033178\times I^{2}-0.84657\times L^{2}$$

The fitting equation for PC is

$$CoF=0.06254-0.070504\times \mu +0.09423\times I+0.56778\times L+0.0010636\times \mu \times I+0.01021\times I\times L\;-0.037791\times \mu \times L+0.0065086\times \mu^{2}-0.097028\times I^{2}-1.6131\times L^{2}$$

The fitting equation for PLA is

$$CoF=0.06853-0.049872\times \mu +0.013833\times I+0.39525\times L+0.00084077\times \mu \times I+0.00020035\times I\times L\;+0.030923\times \mu \times L+0.004864\times \mu^{2}-0.025848\times I^{2}-1.1626\times L^{2}$$

In Figure 7, the empirical models for ABS, PC, and PLA yield R-squared values of 0.992, 0.959, and 0.956, respectively, demonstrating a high degree of goodness of fit. Correspondingly, the Root Mean Square Error (RMSE) values are calculated as 0.00667 for ABS, 0.00847 for PC, and 0.00673 for PLA. These robust R-squared values, all exceeding 0.95, underscore the models’ credibility.

A closer examination of the derived equations reveals that among the three independent variables—layer thickness, filler density, and material type—layer thickness exerts the most significant influence on the friction coefficient. Additionally, it is noteworthy that the combined effect of the layer thickness and filler density on the friction coefficient is notably modest. Importantly, the observed variations in the friction coefficient align closely with the predictions made by these equations.

Subsequently, each material is individually modelled with its unique coefficients. These coefficients are later employed as dependent variables, subjected to curve-fitting concerning the respective shear modulus for each material. This procedure enables the development of empirical models for the surface friction coefficient, encompassing different materials, lubrication modes, layer thicknesses, and filler densities. The specific coefficients are comprehensively tabulated in Table 3, wherein “*G*” denotes the shear modulus of the respective material under consideration.

Following the establishment of the empirical model, this investigation opted to employ PC-ABS as the material of choice to validate the model’s reliability. Six sets of specimens were prepared for the validation test, wherein the parameters for the independent variables were intentionally varied from those employed in the original empirical model, except for the lubrication mode, which remained consistent. A comprehensive presentation of these specific parameters and their corresponding outcomes is delineated in Table 4.

**Figure 8:**
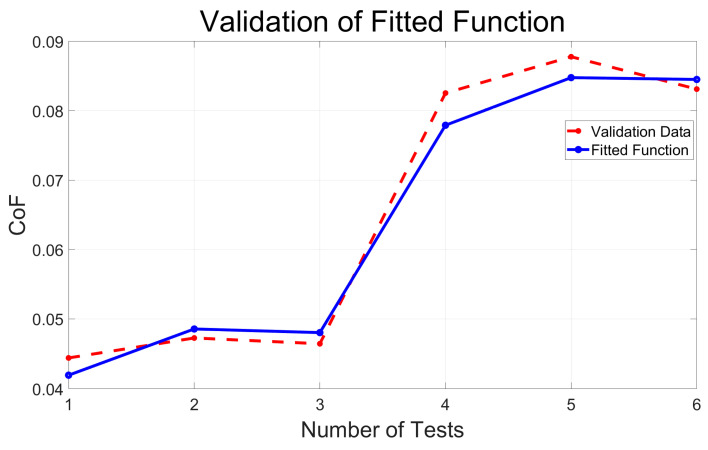
Comparison of validation and model prediction results for the CoF.

The CoF acquired using experimentation has been compared with the predicted values calculated by the empirical model. The outcomes of this comparison are graphically illustrated in Figure 8. It is discernible from these results that the experimental findings closely mirror the projected trends in the model. To quantify the model’s accuracy, we calculate the RMSE to be 0.0027, along with an R-squared value of 0.9809. These metrics collectively signify high precision and reliability in the empirical model devised in this study.

## 5. Conclusions

This study comprehensively investigated the tribological properties of 3D-printed material surfaces, examining the influence of various factors such as material type, lubrication mode, layer thickness, and infill density using a thorough full-factor analysis. It introduced an innovative approach by incorporating different materials and lubrication modes into the model-building process, unifying empirical models to predict the CoF.

The study found that the CoF for ABS, PC, and PLA materials exhibited a typical pattern of increasing and decreasing with varying layer thicknesses and infill densities, peaking at 0.2 mm thickness and 50% infill. The impact of lubrication modes on the surface friction coefficients was observed, with the most pronounced effect on ABS, followed by PC and PLA. Notably, oil lubrication and low-viscosity lubricants effectively reduce material wear.

A non-linear empirical model was proposed to predict the CoF. The model’s reliability was validated using the PC-ABS material, demonstrating a high R-squared value of 0.9809.

## Figures and Tables

**Table 1:** Material mechanical properties.

Material	ABS [28]	PC [29]	PLA [30]	PC-ABS [29]
Tensile strength (MPa)	33.3	62.7	46.6	37.2
Shear modulus (GPa)	2	2.35	2.65	2.2
Strength (MPa)	59.0	100.4	85.1	66.3
Density (g/cm^3^)	1.12	1.19	1.17–1.24	1.1

**Table 2:** Independent variables and their values in the experiment.

Parameters	Value		
Material	ABS	PC	PLA
Layer Thickness (mm)	0.1	0.2	0.25
Infill Density (%)	25	50	75
Lubrication Modes	None	Grease	Oil

**Table 3:** Expressions for the coefficients in the nonlinear fitting equation.

$p_{000}$	$0.5214\times G^{2}-2.587\times G+3.2626$	$p_{101}$	$-0.0942\times G^{2}+0.4375\times G-0.4979$
$p_{001}$	$-0.1259\times G^{2}+0.6983\times G-1.0162$	$p_{110}$	$1.0052\times G^{2}-4.7968\times G+5.6837$
$p_{010}$	$-0.6987\times G^{2}+3.2255\times G-3.6272$	$p_{002}$	$0.013\times G^{2}-0.0706\times G+0.1005$
$p_{100}$	$-2.6993\times G^{2}+12.9213\times G-14.8905$	$p_{020}$	$0.6457\times G^{2}-2.9912\times G+3.3664$
$p_{011}$	$-0.0195\times G^{2}+0.097\times G-0.1189$	$p_{200}$	$5.6796\times G^{2}-26.8964\times G+30.2278$

**Table 4:** The validation group and results for the CoF.

Number of Tests	Lubrication	Infill Density	Layer Thickness	Observed CoF	Predicted CoF	Errors
1	Oil	20%	0.22	0.044416	0.04194965	5.879%
2	Oil	40%	0.22	0.047262	0.048552962	2.659%
3	Oil	60%	0.22	0.046442	0.048032114	3.311%
4	Grease	20%	0.16	0.082543	0.077907846	5.950%
5	Grease	40%	0.16	0.087777	0.084769062	3.548%
6	Grease	60%	0.16	0.083115	0.084506118	1.646%

## Data Availability

Data available on request.
